# From fragmented to flawless: A large-scale synthetic micrograph library for benchmarking microstructural image restoration

**DOI:** 10.1016/j.dib.2026.112783

**Published:** 2026-04-24

**Authors:** Nikhil Chaurasia, Shikhar Krishn Jha, Sandeep Sangal

**Affiliations:** Indian Institute of Technology Kanpur, Kanpur, Uttar Pradesh, India

**Keywords:** Quantitative metallography, Computer vision, Artifact removal, Ground truth, Steel

## Abstract

The accurate extraction of grain boundaries from metallographic images is a prerequisite for quantitative microstructural analysis, but it is often hindered by experimental artifacts such as noise, etching pits, scratches, and incomplete boundaries. Manual correction of these defects is a significant bottleneck, being both time-consuming and subjective. This article presents a large-scale, synthetic dataset of 512×512 pixel images designed to train and benchmark deep learning models for two critical automated image processing tasks: noise cleaning and grain boundary reconstruction. The dataset was generated by first creating clean, single-phase polycrystalline micrographs using the polySim library. These base images were then programmatically degraded by adding a combination of real and artificial noise and by introducing discontinuities into the grain boundary network. The final dataset comprises 14,999 paired PNG images, consisting of 5999 pairs for noise removal (degraded vs. clean ground truth) and 9000 pairs for grain boundary reconstruction (fragmented vs. connected networks), enabling the development of robust models for automated, objective, and reproducible microstructural image analysis.

Specifications Table

**Table:** 

Subject	Engineering & Materials science
Specific subject area	Microstructure Denoising, Grain Boundary Reconstruction, Deep Learning
Type of data	Synthetic phase polycrystalline template and the corresponding ground truth (PNG format)
Data collection	Synthetic single-phase micrographs were generated using the polySim Python library [1]. These were then programmatically degraded by adding noise (scratches, pits) and introducing discontinuities to the grain boundaries to create the final raw/processed image pairs.
Data source location	Indian Institute of Technology Kanpur, India
Data accessibility	Data Repository: Figshare (Images): Synthetic Micrograph Data for Denoising and Grain Boundary Reconstruction DOI to Data (Images): https://doi.org/10.6084/m9.figshare.30328510 Direct URL to Code (Generation Scripts Denoising): https://github.com/nikolspace/Denoise Direct URL to Code (Generation Scripts Grain Boundaries): https://github.com/nikolspace/Reconstruction-GB
Related research article	https://doi.org/10.1016/j.mtla.2026.102715

## 1 Value of the Data

• This dataset is a high-value resource for materials informatics researchers developing robust segmentation models, metallurgists seeking to automate grain size analysis, and computer vision scientists specializing in domain-specific image-to-image translation.
• The dataset enables the automation of the tedious and subjective manual process of separating grain boundaries from experimental noise and artifacts, significantly increasing the speed and objectivity of high-throughput microstructural analysis.
• By providing a unique resource for tackling incomplete or broken grain boundaries, the data allows researchers to build and validate novel models that automatically reconstruct a complete grain boundary network from fragmented or discontinuous inputs.
• The dataset serves as a standardized benchmark for the materials science community; its pixel-perfect ground truth allows for direct, quantitative evaluation of new algorithms using metrics like IoU, PSNR, and SSIM.
• The utility of this data has been validated through its successful application in a primary research study [2] published in Materialia, where it served as the foundational training resource for a deep-learning-based framework for grain boundary reconstruction.

## 2 Background

Accurate quantification of grain structure is fundamental to establishing structure-property relationships in materials. However, images obtained from experimental characterization are often imperfect, containing noise and artifacts like etching pits and scratches, or suffering from incomplete grain boundaries due to inconsistent sample preparation. Manual correction of these defects is a major bottleneck in high-throughput analysis. Deep learning models present a powerful solution for automating these image restoration tasks but require large, high-quality datasets of degraded images paired with their ``perfect'' ground truth counterparts. This dataset was compiled to fill this need, providing a comprehensive resource for training and benchmarking such models. This data article adds value to the related research article by describing the foundational dataset and the associated programmatic generation methodology in detail, making it independently discoverable and reusable by the broader scientific community.

## 3 Data Description

The dataset is organized into two primary folders for each task: 1_Noise_Cleaning and 2_Grain_Boundary_Reconstruction. All images are 512×512 pixel PNG files.

**Fig. 1:**
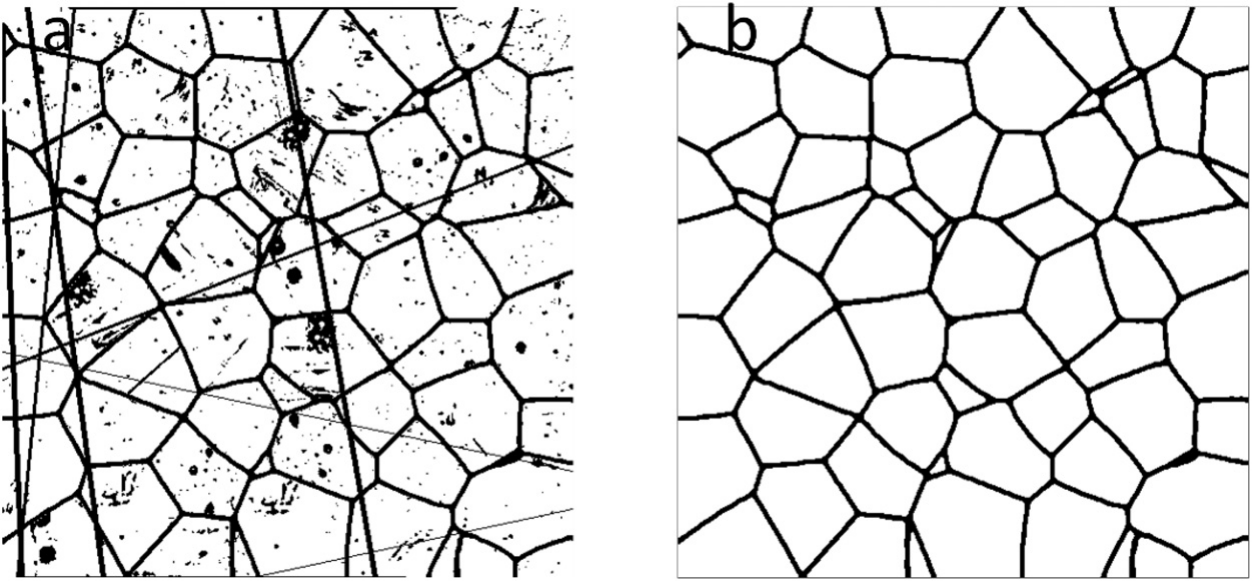
Example image pair from the Noise Cleaning dataset. (a) A synthetic micrograph with programmatically added noise and artifacts (file ‘1.png’). (b) The corresponding clean ground truth image showing only the complete grain boundaries (file ‘1.png’).
• **Grain Boundary Reconstruction Dataset**: Contains 9000 image pairs. Training/ (8000 pairs) and Testing/ (1000 pairs) subfolders contain the input (Broken_Boundaries/) and ground truth (Complete_Ground_Truth/). An example is shown in Fig. 2. For both tasks, a one-to-one correspondence is maintained via identical file naming.

• **Noise Cleaning Dataset**: Contains 5999 image pairs. Training/ (4999 pairs) and Testing/ (1000 pairs) subfolders contain the input (Noisy_Images/) and ground truth (Clean_Ground_Truth/). An example is shown in Fig. 1.

**Fig. 2:**
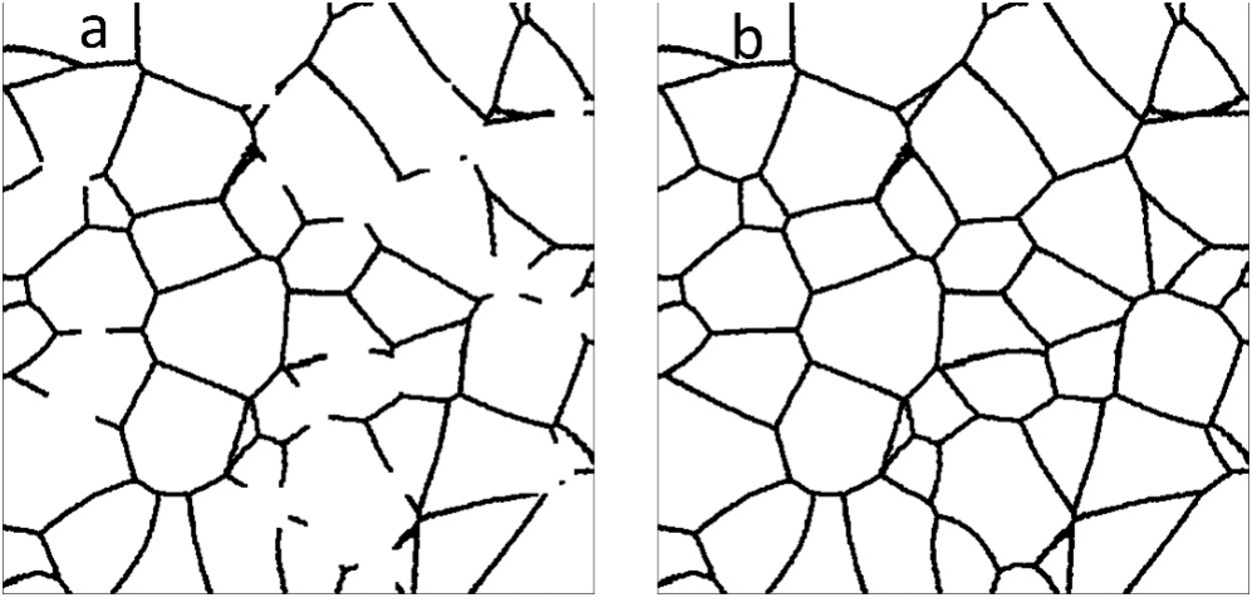
Example image pair from the Grain Boundary Reconstruction dataset. (a) Synthetic micrograph with programmatically fragmented grain boundaries (file ‘0.png’). (b) The corresponding ground truth image showing the fully reconstructed and continuous grain boundary network (file ‘0.png’).

## 4 Experimental Design, Materials and Methods

The methodology utilized in this work is detailed in the associated primary research article [2] and builds upon the framework established in [3]. A concise summary of the experimental and programmatic protocols is provided below.

### 4.1 Reference sample preparation for validation

**Fig. 3:**
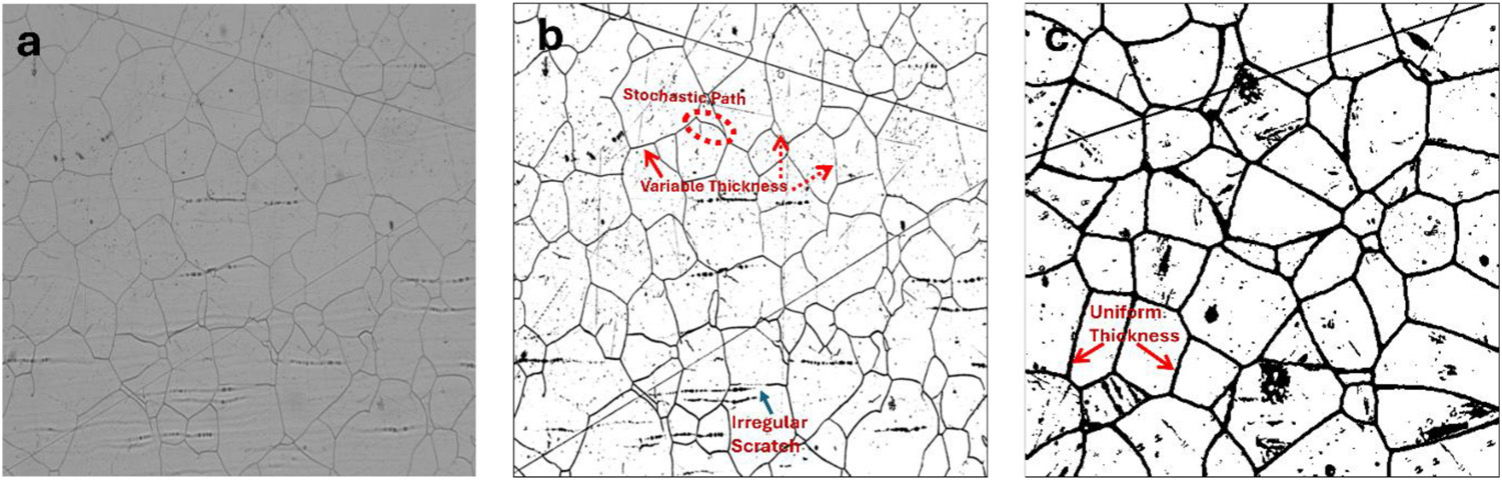
Qualitative validation of realism and domain gap identification. (a) Original experimental micrograph of SS316 steel. (b) Annotated thresholded micrograph highlighting stochastic geometric variations, irregular scratches, and variable boundary thickness. (c) Annotated synthetic Sample 349th showing the domain gap through idealized curvatures and uniform programmatic boundary thickness.

To validate the synthetic dataset, experimental micrographs were obtained from a Stainless Steel 316 (SS316) specimen. The sample was electropolished using a solution of HNO_3_ H2SO_4_, and deionized water (30:40:30 ratio) for 20 s at 20 mA and 12 V. These experimental images provide the baseline for comparing real-world artifact morphologies—such as variable boundary thickness and stochastic geometric variations—against the synthetic data (Fig. 3).

### 4.2 Base micrograph generation

Clean, single-phase, equiaxed grain structures were synthetically generated as 512 times 512 pixel images using the polySim Python library [1]. The simulation parameters were calibrated to produce a diverse range of grain sizes and topologies representative of annealed polycrystalline materials.

### 4.3 Programmatic degradation and dataset organization

The base micrographs were programmatically degraded to create two task-oriented datasets:

• Noise Removal: A combination of real experimental artifacts (cropped etching pits and polishing scratches) and artificial noise functions were overlaid onto the clean base micrographs using the denoise_data_generation.py script.
• Boundary Reconstruction: Grain boundary discontinuities were introduced using the generate_data.py script to simulate incomplete etching. Both scripts and the organized dataset are available in the public GitHub repositories listed in the Specifications Table.

### 4.4 Technical utility

The technical utility of the library was validated using an Attention U-Net and UNET evaluated on an unbiased test set of 1000 synthetic micrographs per task. As summarized in Table 1, the model achieved a mean Intersection over Union (IoU) of 0.993 ± 0.004 for noise removal and 0.96 ± 0.02 for grain boundary reconstruction. Full details regarding the model architecture and performance analysis are documented in the associated research article [2].

**Table 1:** Baseline performance metrics for microstructural restoration.

Task	IoU
Denoising	0.993 ± 0.004
Grain Boundary Reconstruction	0.96 ± 0.02

## Limitations

While this dataset provides a standardized benchmark with pixel-perfect ground truth, it is primarily limited by the synthetic-to-real domain gap in artifact morphology and topological representation. A qualitative comparison (Fig. 3) between synthetic Sample 349th from the training dataset and experimental SS316 steel optical micrograph reveals that programmatically generated grain boundaries exhibit constant thickness and idealized curvatures. In contrast, real micrograph demonstrate significant variability in boundary thickness and stochastic geometric variations. Furthermore, the dataset's morphological scope is restricted to single-phase, equiaxed structures; it does not account for the complex textures of rolled materials, highly deformed grains, or twin microstructures frequently encountered in experimental microscopy.

## Ethics Statement

The authors have read and follow the ethical requirements for publication in *Data in Brief*. The current work does not involve human subjects, animal experiments, or any data collected from social media platforms.

## CRediT Author Statement

**Nikhil Chaurasia**: Conceptualization, Methodology, Software, Data Curation, Writing – Original Draft; **Shikhar Krishn Jha**: Supervision, Conceptualization, Review & Editing, Investigation; **Sandeep Sangal**: Supervision, Conceptualization, Writing – Review & Editing.

## Data Availability

figshareSynthetic Micrograph Data for Denoising and Grain Boundary Reconstruction (Original data). githubReconstruction-GB: Programmatic Methodology for Synthetic Micrograph Generation (Reference data). GithubDenoising-GB: Hybrid Microstructural Noise Cleaning Framework (Reference data).

## References

[1] N. Chaurasia, S. Sangal, S.K. Jha, polySim: polycrystalline microstructure Simulation package v0.1.1, 2024. https://pypi.org/project/polySim/.

[2] Chaurasia N., Jha S.K., Sangal S. (2026). Noise removal and grain-boundary reconstruction in single-phase polycrystalline microstructures using deep learning. Materialia.

[3] Chaurasia N., Jha S.K., Sangal S. (2023). A novel training methodology for phase segmentation of steel microstructures using a deep learning algorithm. Materialia.

